# Potential Years of Life Lost Due to COVID-19 in the United States, Italy, and Germany: An Old Formula with Newer Ideas

**DOI:** 10.3390/ijerph17124392

**Published:** 2020-06-18

**Authors:** Amal K. Mitra, Marinelle Payton, Nusrat Kabir, April Whitehead, Kimberly N. Ragland, Alexis Brown

**Affiliations:** Department of Epidemiology and Biostatistics, Jackson State University, School of Public Health, Jackson, MS 39213, USA; marinelle.payton@jsums.edu (M.P.); nusratkabir14@gmail.com (N.K.); aprilwhitehead96@gmail.com (A.W.); kimberly.raglandjones@gmail.com (K.N.R.); J00889135@students.jsums.edu (A.B.)

**Keywords:** COVID-19, PYLL, disease burden, United States, Italy, Germany, New York State

## Abstract

Today, the world is facing the challenge of a major pandemic due to COVID-19, which has caused more than 6.1 million cases of infection and nearly 370,000 deaths so far. Most of the deaths from the disease are clustered in the older population, but the young and children are not spared. In this context, there is a critical need to revisit the formula for calculating potential years of life lost (PYLL). Data on age-specific deaths due to COVID-19 in three countries, including the United States (US), Italy, and Germany, were evaluated. New York State, as a significant outlier within the US, was also included. PYLLs in the US were five times as high as those of Italy. Compared with Germany, PYLLs in Italy were 4 times higher, and the rates in the US were 23, 25, and 18 times higher when using upper age limits of 70, 75, and 80, respectively. Standardized PYLLs in New York were 2 times as high as the rates in Italy, and 7 to 9 times as high as PYLLs in Germany. The revised formula of PYLL, using an upper limit of age 80, is recommended to accurately measure premature deaths due to a major disastrous disease such as COVID-19.

## 1. Introduction

The current pandemic of COVID-19 has created havoc by affecting 213 countries and territories. Every day, the incidence and death rates due to COVID-19 have been escalating in an exponential trend [1]. Obviously, this pandemic has suddenly made a paradigm shift in our existing knowledge of the global burden of diseases. Due to the changing pattern of risk due to COVID-19, with 60–70% of mortality being observed in the elderly population [2,3,4,5], this pandemic raises questions among researchers when using some of the conventional methods of assessing the societal burden of diseases. The common metrics used for quantifying the overall disease burden and prioritizing causes of deaths are disability-adjusted life years (DALYs), potential years of life lost (PYLL), quality-adjusted life-year (QALY), and disability-adjusted life expectancy (DALE), among others [6,7,8,9].

PYLL is often used in calculating deaths and comparing the health system performance of countries in addressing major killer diseases such as cancer, cerebrovascular disease, ischemic heart disease, accidents, infectious diseases, and others [10,11,12]. PYLL, as proposed by Romeder and McWhinnie in 1977, is a measure of premature mortality [12]. The method of estimating PYLL involves calculating the additional time a person would have lived had he or she not died prematurely [12]. In this method, greater weight is given to deaths at a younger age and a lower weight to deaths at an older age. Thus, PYLL emphasizes the loss of the potential contribution that younger individuals could make to society [12]. Interestingly, the COVID-19 pandemic caused a paradigm shift of our knowledge because deaths from this pandemic disproportionately affected the elderly population [2,3,4,5,13,14,15]. In this context, the fundamental science behind using more weight for the loss of younger aged people and discounting the loss due to the deaths of older people in the case of PYLL is under challenge. Additionally, applying the conventional formula of PYLL in the case of COVID-19 is misleading and is an underestimation of the actual societal loss in terms of potential years of life should the people lived longer. Therefore, there is a critical need to address fundamental and important questions regarding the calculation of PYLL based on the concept that people are living longer and can contribute more years to society.

In the original formula of PYLL [12] in 1977, age 70 was considered the upper age limit. The Organization for Economic Co-operation and Development (OECD), an intergovernmental economic organization with 36 member countries, including the most economically developed countries, has suggested using the age of 75 for the calculation of PYLL [16]. The Association of Public Health Epidemiologists in Ontario, Canada, has also been using age 75 as the upper limit since 2006, based on an average life expectancy of Canadians for both sexes combined [17].

However, in Canada, the average life expectancy is 80 years for males and 84 years for females in 2019 [18]. According to the World Bank Report [18], the average life expectancy at birth for most countries of the European Union is 80 years or more: Austria—82 years, Belgium—82 years, Denmark—81 years, France—83 years, Germany—81 years, Italy—83 years, Netherlands—82 years, Spain—83 years, and Sweden—83 years. The average life expectancy in the United States was 79 years in 2019. Because of the increase in the life expectancy in recent years in most developed (and developing) countries, and changes in disease patterns even in the case of infectious diseases such as COVID-19, the overall objective of this research is to develop a new but practical and scientific method in the calculation of societal loss by using PYLL. The specific aim of this study is to compare three upper age limits for the calculation of PYLL in different countries to better represent the societal loss due to deaths from COVID-19. The selection of countries for this study was based on the availability of data on age-specific number of deaths, which is required for the calculation of PYLL [12].

## 2. Materials and Methods

### 2.1. Data Acquisition and Validation

Secondary data on COVID-19 cases and deaths were obtained from multiple sources, including websites of Worldometer [1], World Health Organization [19], Johns Hopkins University Coronavirus Resource Center [20], Centers for Disease Control and Prevention (CDC) [21], Statista Research Department [22], New York State Department of Health [23], search engines such as PubMed and MEDLINE—two US National Library of Medicine premier bibliographic databases, and Google Scholar. The current data on life expectancy was obtained from the World Bank Report [18]. Country-specific population data were extracted from the United Nations Population Division estimates of 2020 [24] and the World Population Review [25].

### 2.2. Selection of Population

Based on records of cases, deaths, and case fatality ratios (CFRs; Table 1), the following countries were initially selected: the US, China, Spain, Italy, France, and Germany. The latest data were extracted until 30 May 2020. Countries having data for age-specific number of deaths, which is the most important component for calculating PYLL, were included in the analyses. Three countries (China, Spain, and France) were excluded because of the unavailability of the required data at the time of the study. In addition to the US, Italy, and Germany, New York State, being the most severely impacted state within the US, was analyzed separately and compared with that of other countries.

### 2.3. Calculation of Age-Specific Mortality and PYLL

Age-specific mortality rates were calculated by dividing the number of deaths in each age group by the total deaths of all ages. The formula for calculating PYLL, as proposed by Romeder and McWhinnie in 1977 is as follows [12]:

$$PYLL=\sum_{i=1}^{69}a_{i}d_{i}=\sum_{i=1}^{69}(70-i-0.5)*d_{i}$$

In the above formula, *a*_i_ = remaining years of life until the upper limit of age (in this case, 70 years); *d*_i_ = number of observed deaths in each class interval; *i* = the mid-point of the class interval of each age group, and 0.5 is a constant.

First, the remaining years of life were calculated for each age group, by subtracting the mid-point of class interval and the constant number of 0.5 from the upper age limit. For example, the mid-point of the class interval of 1–4 years is 2.5. Taking age 70 as the upper limit, the remaining years of life = 70 − 2.5 − 0.5 = 67.

PYLLs were calculated for each age group and for the three upper age limits of 70, 75, and 80 years. The selection of ages 70 and 75 years was based on previous reports [16,17]. In this study, age 80 was used as a new upper age limit based on the current life expectancy in most developed countries [18]. The total PYLL is the summation of all age-specific PYLLs.

### 2.4. PYLL Rates and Standardization

PYLL rates for the three upper age limits were calculated using the population of the country (or the state population for New York) as the denominator. For standardization, the population of Italy, being the lowest of the three-country populations, was selected as the reference in this study. All rates were expressed per 100,000. Any methodological issues were discussed.

## 3. Results

***Italy***: As of 30 May 2020, there were 232,664 cases and 33,340 deaths due to COVID-19, yielding a CFR of 14.33% in Italy (Table 1). About 85% of all deaths due to COVID-19 occurred among people aged 70 and above (Table 2). Only 1.1% of the deaths occurred at ages less than 50. The country lost a large number of potential years due to COVID-19—a total of 42,560, 66,070, and 132,260 person-years before the population reached age 70, 75, and 80 years, respectively. The highest number of PYLL was in the age group of 60–69 years.

**Figure 1:**
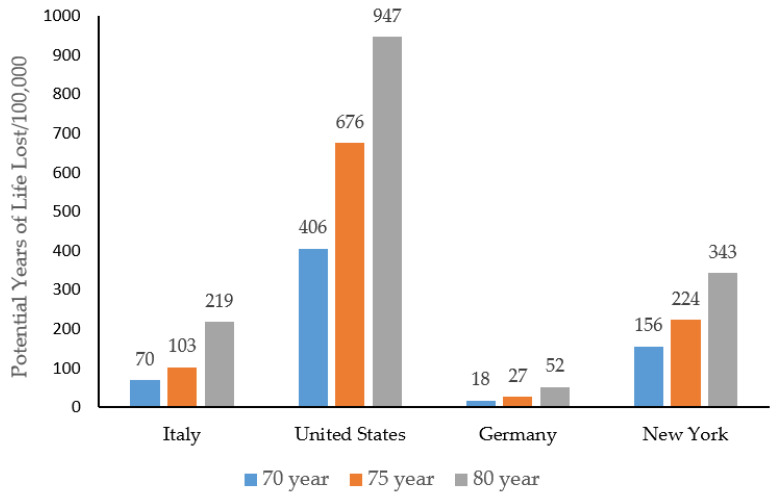
Comparison of potential years of life lost (PYLL) due to COVID-19 in the study populations.

***The United States***: The US COVID-19 cases rose to more than 1.8 million, with more than 105,000 deaths as of 30 May 2020. The CFR of Italy was 2.5 times higher than that of the US (Table 1). Of the 81,372 deaths having age distribution available in the US (Table 3), more than 80% were 65 years or older, and only 2.5% were younger than age 45. PYLL due to COVID-19 in the US surpassed any other countries. The total PYLL due to COVID-19 was 245,246, 408,781, and 572,316 person-years before reaching age 70, 75, and 80 years, respectively. When the total PYLL figures were compared, the person-year loss in the US was 6 times higher than those of Italy for the age limits of 70 and 75, but about 4 times higher when age 80 was used as the upper limit. The standardized PYLL measurements provided similarly higher rates in the US compared with Italy (Figure 1). However, the crude PYLLs before standardization in the US were only 4 units (person-years/100,000) higher at age 70, 14 units higher at age 75, and 46 units lower at age 80, compared with those of Italy, indicating that standardized rates provide more meaningful information for cross-country comparisons.

In this figure, ages 70, 75, and 80 years were used as the upper limit. The population of Italy was used as a reference. The PYLL rates for the US, Germany, and New York were standardized based on the population of Italy.

***Germany***: Among the countries considered in this study, Germany had the lowest deaths and CFR (4.69%; Table 1). Similar to the other countries, a higher death rate was observed in older age groups. The total PYLL was lower in Germany compared to other countries (Table 4). When standardized PYLL rates were used, the PYLL rates in Italy were approximately 4 times higher, and the rates in the US were 23, 25, and 18 times higher at age 70, 75, and 80 years, respectively, compared to those of Germany.

***New York State***: Table 5 shows only 23,848 COVID-19 deaths with age-group distributions reported by the State Department of Health in New York [23] as of 29 May 2020. Of them, the highest death rates were in the age group 70 to 89 years. Deaths in the younger ages below age 50 were about 5% of the total. The highest number of PYLL was in the age-group of 60–69 years, where total years of life lost were 23,350, 46,700, and 70,050 person-years for 70, 75, and 80 years of age, respectively. The standardized rates of PYLL in New York were approximately 2 times as high as the rates in Italy. Similarly, compared to the PYLLs in Germany, the rates in New York were 9, 8, and 7 times higher at age 70, 75, and 80 years, respectively.

## 4. Discussion

This is the first study to demonstrate life-expectancy-adjusted PYLL in estimating the burden of mortality and a societal loss of manpower in a major disaster such as COVID-19. This study validates the importance of measuring PYLL across populations in a disastrous situation where many people die prematurely. In addition, this study validates that the standardized measurement of PYLL is essential when the measurement is applied to compare across countries or different populations [16].

### Methodological Issues in Using the Upper Age Limit

It is noteworthy that in all of the case studies presented in this study, a similar pattern was observed—the person-year lost in the older age groups was not considered in calculating PYLL in some of the upper age limits such as 70 years and 75 years. For example, the person-year lost at age group 70–79 was not considered in the calculation of PYLL when the upper age limits of 70 and 75 were used, but it was not the case when age 80 was used as the upper limit. This was true in the case of Italy, Germany, and New York State. Although the reported age-groups were slightly different in the US compared to the other populations in the study, the pattern remained the same. In the US, the person-year lost in the older ages, such as in age-group 65–74 years, was nullified at the upper limit of age 70 but not so in the other two upper age limits of 75 and 80. These issues of calculating PYLL are important to consider because COVID-19 deaths occurred most commonly in the older age groups [2,3,4,5].

Of the three age limits used in this study, choosing a cut-off age of 70 is obsolete based on the current state of life expectancy in most populations [18]. Our data showed that the use of age 80 as the upper limit for PYLL calculations is probably more reasonable when the other two groups, ages 70 years and 75 years were compared. For example, deaths at ages 70 years and above had no additional contributions toward the calculation of PYLL at the upper limits of ages 70 and 75. While deaths due to COVID-19 were highest in the age group 70 years and above, as is consistent with other studies [2,3,4], a complete elimination of the person-year loss in the older population may not provide a valid picture of the total PYLL in the context of this pandemic. The imprecise estimation of PYLLs and disability-adjusted life years (DALYs) are especially significant because these metrics are often used to determine resource allocation and health policy decision-making [22]. The rationale for using age 80 was based on the current life expectancy at birth in most of the developed countries [18]. However, as life expectancy increases, a further study may be justifiable, using another upper age limit (such as 85 or more) in the future. In addition to using age 80 as the upper limit in the context of developed countries, a standardized rate is recommended for cross-country comparisons [10,16]. This research project will set new paradigms in the quantification of premature deaths in terms of person-years lost.

In a recent study [26], Kirigia and Muthuri (2020) reported the economic impact of deaths due to COVID-19 in China. The total deaths in China was 2595, as of 24 February 2020. The total fiscal value of the COVID-19 deaths in that country was Int$ (International dollars) 924,346,795. The average fiscal value per death was Int$ 356,203. If we want to translate the US deaths due to COVID-19 in terms of economic loss, that figure would be enormous.

The lack of consistency in age breakdown [21,22,23] and reporting of age-specific death rates instead of the number of deaths by age [22] are identified as important methodological issues. Due to these issues, the data of China, Spain, and France could not be used in the analyses. However, the primary goal of this study is to evaluate whether PYLL can be used as an indicator of societal loss in the case of a major pandemic. That goal was met. Still, it would have been more convincing if we could have shown the application of the parameter using more country-level data. The case of New York State [23], however, offered at least another population where PYLL was applicable to assessing the societal loss. We advocate that a similar study should be done in other populations to assess a greater picture of the loss due to the COVID-19 pandemic and also to show the consistencies. Furthermore, for developing countries where life expectancies are lower than developed countries, studies are needed to determine a reasonable cut-off point of the upper age limit.

The criticisms that pertain to PYLL calculations have been adequately addressed in this research: (1) the use of different age weightings for PYLL at different ages has been minimized in this study, (2) the discounting mechanism of PYLL, by assigning a new upper age limit based on the current life expectancy-value, is adjusted, and (3) the use of PYLL in an epidemic situation has been established.

*Limitations*. One important limitation of this study was that the pandemic is still ongoing, and new data are being generated on a daily basis. The current mortality data in this study may not reflect the latest data and the exact gravity of the problem of the pandemic. The calculated PYLL rates are an underestimation of the real figures because of the rising number of deaths due to the pandemic. Another challenge in standardization was the lack of information about the age-specific population in the reference population. However, using the reference population as the denominator was shown to be a better alternative when comparing the rates across countries.

*Policy Implications*. Based on the current figure of life expectancy at birth in most developed countries, an upper limit age of 80 years is more realistic. A different age limit should be used when PYLL is measured for developing countries. Alternatively, the exact figure for life expectancy in each country may be used as the age limit. In that case, the comparability of data across countries could be an issue. More country-specific data are needed on the COVID-19 pandemic for international comparison of health system performances amongst countries and more accurate assessment of premature life lost due to the pandemic. A follow-up study would be useful for the aftermath of premature life lost once the pandemic is over. When addressing the issue of societal loss in general, several other factors in this analysis, which were not accounted for due to the scope of this study, could be considered in selecting populations. In future studies, perhaps differences in urban versus rural, healthcare outcomes, total healthcare system capacity/burden, and other population health measures such as obesity and chronic diseases could be investigated.

## 5. Conclusions

Based on our study findings, the revised formula, using an upper age limit of 80, measured the societal loss in terms of PYLL more precisely, as compared to using ages 70 and 75 as the upper limit. The findings of this study suggest that a standardized PYLL, using a reference population, is a better measure of premature life lost when cross-country data are compared. The research is innovative, and it can be applied in a disaster situation such as COVID-19. The revised formula should be further applied in assessing health system performances of countries when addressing major killer diseases, and in assessing risk and potential health needs in any future epidemic conditions.

## Figures and Tables

**Table 1:** Cases, deaths, and case fatality ratios (CFRs) in countries most affected by the COVID-19 pandemic, as of 30 May 2020.

Country	Cases	Deaths	CFR (%)
United States	1,811,102	105,337	5.82
Spain	286,308	27,125	9.47
Italy	232,664	33,340	14.33
France	188,625	28,771	15.25
Germany	183,149	8598	4.69
World	6,111,187	369,376	6.04

Source: https://www.worldometers.info/coronavirus/ [1].

**Table 2:** Potential years of life lost (PYLL) due to COVID-19 in Italy, as of 25 May 2020.

Age Group (year)	Mid-Point of Class Interval (*i*)	COVID-19 Deaths (*d*) *	PYLL at Age Limit		
			70 Years	75 Years	80 Years
0–9	4.5	4 (0.01)	260	280	300
10–19	14.5	0	0	0	0
20–29	24.5	12 (0.04)	540	600	660
30–39	34.5	62 (0.20)	2170	2480	2790
40–49	44.5	272 (0.86)	6800	8160	9520
50–59	54.5	1103 (3.50)	16,545	22,060	27,575
60–69	64.5	3249 (10.30)	16,245	32,490	48,735
70–79	74.5	8536 (27.06)	0	0	42,680
80–89	84.5	12,926 (40.98)	0	0	0
90+		5382 (17.06)	0	0	0
Total		31,546 (100.0)	42,560	66,070	132,260
PYLL/100,000 ^†^			70.39	109.28	218.75

* Source: https://www.statista.com/statistics/1105061/coronavirus-deaths-by-region-in-italy/ [22]. ^†^ The denominator is the population of Italy = 60,461,826 [24].

**Table 3:** Potential years of life lost (PYLL) due to COVID-19 in the United States as of 28 May 2020.

Age Group (Year)	Mid-Point of Class Interval (i)	COVID-19 Deaths (*d*) *	PYLL at Age Limit		
			70 Years	75 Years	80 Years
<1	0.5	5 (0.006)	345	370	395
1–4	2.5	3 (0.004)	201	216	231
5–14	9.5	11 (0.01)	660	715	770
15–24	19.5	93 (0.11)	4650	5115	5580
25–34	29.5	542 (0.67)	21,680	24,390	27,100
35–44	39.5	1403 (1.72)	42,090	49,105	56,120
45–54	49.5	3893 (4.78)	77,860	97,325	116,790
55–64	59.5	9776 (12.01)	97,760	146,640	195,520
65–74	69.5	16,981 (20.87)	0	84,905	169,810
75–84	79.5	21,822 (26.82)	0	0	0
85+	89.5	26,843 (32.99)	0	0	0
Total deaths		81,372 (100.0)			
Total PYLL			245,246	408,781	572,316
PYLL/100,000 ^†^			74.09	123.50	172.90
PYLL/100,000, standardized ^‡^			405.62	676.10	946.57

* Source: https://www.cdc.gov/nchs/nvss/vsrr/covid_weekly/index.htm#AgeAndSex [21]. ^†^ The denominator is the US population = 331,002,651 [24]. ^‡^ The denominator is the population of Italy (reference) = 60,461,826 [24].

**Table 4:** Potential years of life lost (PYLL) due to COVID-19 in Germany as of 27 May 2020.

Age Group (Year)	Mid-Point of Class Interval (*i*)	COVID-19 Deaths (*d*) *	PYLL at Age Limit		
			70 Years	75 Years	80 Years
0–9	4.5	1 (0.01)	65	70	75
10–19	14.5	2 (0.02)	110	120	130
20–29	24.5	8 (0.10)	360	400	440
30–39	34.5	20 (0.24)	700	800	900
40–49	44.5	62 (0.75)	1550	1860	2170
50–59	54.5	283 (3.43)	4245	5660	7075
60–69	64.5	767 (9.29)	3835	7670	11,505
70–79	74.5	1851 (22.43)	0	0	9255
80–89	84.5	3704 (44.89)	0	0	0
90–99	94.5	1504 (18.23)	0	0	0
100+		50 (0.61)	0	0	0
Total		8252 (100)	10,865	16,580	31,550
PYLL/100,000 ^†^			12.97	19.79	37.66
PYLL/100,000, standardized ^‡^			17.97	27.42	52.18

* Source: https://www.statista.com/statistics/1105512/coronavirus-covid-19-deaths-by-gender-germany/ [22]. ^†^ The denominator is the population of Germany = 83,783,942 [24]. ^‡^ The denominator is the population of Italy (reference) = 60,461,826 [24].

**Table 5:** Potential years of life lost (PYLL) due to COVID-19 in New York State as of 29 May 2020.

Age Group (Year)	Mid-Point of Class Interval (*i*)	COVID-19 Deaths (*d*) *	PYLL at Age Limit		
			70 Years	75 Years	80 Years
0–9	4.5	4 (0.02)	260	280	300
10–19	14.5	10 (0.04)	550	600	650
20–29	24.5	88 (0.37)	3960	4400	4840
30–39	34.5	316 (1.33)	11,060	12,640	14,220
40–49	44.5	836 (3.51)	20,900	25,080	29,260
50–59	54.5	2279 (9.56)	34,185	45,580	56,975
60–69	64.5	4670 (19.58)	23,350	46,700	70,050
70–79	74.5	6204 (26.01)	0	0	31,020
80–89	84.5	6131 (25.71)	0	0	0
90+		3301 (13.84)	0	0	0
Unknown		9 (0.04)	0	0	0
Total		23,848 (100)	94,265	135,280	207,315
PYLL/100,000 ^†^			484.89	695.87	1066.41
PYLL/100,000, standardized ^‡^			155.91	223.74	342.89

* Source: https://covid19tracker.health.ny.gov/views/NYS-COVID19-Tracker/NYSDOHCOVID-19Tracker-Fatalities?%3Aembed = yes&%3Atoolbar = no&%3Atabs = n [23]. ^†^ The denominator is the population of New York State = 19,440,469 [25]. ^‡^ The denominator is the population of Italy (reference) = 60,461,826 [24].

## References

[1] Worldometer COVID-19 Coronavirus Pandemic. https://www.worldometers.info/coronavirus/.

[2] The Novel Coronavirus Pneumonia Emergency Response Epidemiology Team (2020). The Epidemiological Characteristics of an Outbreak of 2019 Novel Coronavirus Diseases (COVID-19)—China, 2020. China CDC Wkly..

[3] Peer N.C., Shrestha N., Rahman M.S., Zaki R., Tan Z., Bibi S., Baghbanzadeh M., Aghamohammadi N., Zhang W., Haque U. (2020). The SARS, MERS and novel coronavirus (COVID-19) epidemics, the newest and biggest global health threats: What lessons have we learned?. Int. J. Epidemiol..

[4] Onder G., Rezza G., Brusaferro S. (2020). Case-fatality rate and characteristics of patients dying in relation to COVID-19 in Italy. JAMA.

[5] Yang X., Yu Y., Xu J., Shu H., Xia J., Liu H., Wu Y., Zhang L., Yu Z., Fang M. (2020). Clinical course and outcomes of critically ill patients with SARS-CoV-2 pneumonia in Wuhan, China: A single-centered, retrospective, observational study. Lancet Respir. Med..

[6] World Health Organization Metrics: Disability-Adjusted Life Year (DALY). https://www.who.int/healthinfo/global_burden_disease/metrics_daly/en/.

[7] Grandjean P., Bellanger M. (2017). Calculation of the disease burden associated with environmental chemical exposures: Application of toxicological information in health economic estimation. Environ. Health.

[8] Egunsola O., Raubenheimer J., Buckley N. (2019). Variability in the burden of disease estimates with or without age weighting and discounting: A methodological study. BMJ Open.

[9] Vienonen M.A., Jousilahti P.J., Mackiewicz K., Oganov R.G., Pisaryk V.M., Denissov G.R., Nurm U., Pudule I., Gurevicius R.J., Zabocki B.M. (2019). Preventable premature deaths (PYLL) in northern dimension partnership countries 2003-13. Eur. J. Public Health.

[10] Canadian Institute for Health Information Potential Years of Life Lost: International Comparisons. Examining Canada’s Health System Performance. https://www.cihi.ca/en/health-system-performance/performance-reporting/international/pyll.

[11] Maximova K., Rozen S., Springett J., Stachenko S. (2016). The use of potential years of life lost for monitoring premature mortality from chronic diseases: Canadian perspectives. Can. J. Public Health.

[12] Romeder J.M., McWhinnie J.R. (1977). Potential years of life lost between ages 1 and 70: An indicator of premature mortality for health planning. Int. J. Epidemiol..

[13] Wu Z., McGoogan J.M. (2020). Characteristics of and important lessons from the coronavirus disease 2019 (COVID-19) outbreak in China. Summary of a report of 72,314 cases from the Chinese Center for Disease Control and Prevention. JAMA.

[14] Remuzzi A., Remuzzi G. (2020). COVID-19 and Italy: What next?. Lancet.

[15] Porcheddu R., Serra C., Kelvin D., Kelvin N., Rubino S. (2020). Similarity in case fatality rates (CFR) of COVID-19/SARS-COV-2 in Italy and China. J. Infect. DEV Ctries.

[16] Organization for Economic Co-Operation and Development (OECD) OECD Health Statistics 2019: Definitions, Sources, and Methods. Potential Years of Life LOST by ICD categories. http://www.oecd.org/health/health-data.htm.

[17] Association of Public Health Epidemiologists in Ontario Calculating Potential Years of Life Lost (PYLL). http://core.apheo.ca/index.php?pid=190.

[18] World Bank Life Expectancy at Birth. https://data.worldbank.org/indicator/SP.DYN.LE00.IN?view=chart.

[19] World Health Organization Rolling Updates on Coronavirus Disease (COVID-19). https://www.who.int/emergencies/diseases/novel-coronavirus-2019/events-as-they-happen.

[20] Johns Hopkins University Coronavirus Resource Center. https://coronavirus.jhu.edu/map.html.

[21] Centers for Disease Control and Prevention National Vital Statistics System. Provisional Death Counts for Coronavirus Disease (COVID-19). https://www.cdc.gov/nchs/nvss/vsrr/covid_weekly/index.htm#AgeAndSex.

[22] Statista Research Department. https://www.statista.com/statistics/1105061/coronavirus-deaths-by-region-in-italy/.

[23] New York State Department of Health Fatalities by Age Group. https://covid19tracker.health.ny.gov/views/NYS-COVID19-Tracker/NYSDOHCOVID-19Tracker-Fatalities?%3Aembed=yes&%3Atoolbar=no&%3Atabs=n.

[24] United Nations Population Division Estimates (2020). Countries in the World by Population. https://www.worldometers.info/world-population/population-by-country/.

[25] World Population Review New York Population 2020 (Demographics, Maps, Graphs). https://worldpopulationreview.com/states/new-york-population/.

[26] Kirigia J.M., Muthuri R.N.D.K. (2020). The fiscal value of human lives lost from coronavirus disease (COVID-19) in China. BMC Res. Notes.

